# Ten simple rules for responsible referencing

**DOI:** 10.1371/journal.pcbi.1006036

**Published:** 2018-04-12

**Authors:** Bart Penders

**Affiliations:** Maastricht University, Care and Public Health Research Institute (CAPHRI), Department of Health, Ethics & Society, Maastricht, the Netherlands; Dassault Systemes BIOVIA, UNITED STATES

We researchers aim to read and write publications containing high-quality prose, exceptional data, arguments, and conclusions, embedded firmly in existing literature while making abundantly clear what we are adding to it. Through the inclusion of references, we demonstrate the foundation upon which our studies rest as well as how they are different from previous work. That difference can include literature we dispute or disprove, arguments or claims we expand, and new ideas, suggestions, and hypotheses we base upon published work. This leads to the question of how to decide which study or author to cite, and in what way.

Writing manuscripts requires, among so much more, decisions on which previous studies to include and exclude, as well as decisions on how exactly that inclusion takes place. A well-referenced manuscript places the authors’ argument in the proper knowledge context and thereby can support its novelty, its value, and its visibility. Citations link one study to others, creating a web of knowledge that carries meaning and allows other researchers to identify work as relevant in general and relevant to them in particular.

On the one hand, citation practices create value by tying together relevant scientific contributions, regardless of whether they are large or small. In the process, they confer or withhold credit, contributing to the relative status of published work in the literature. On the other hand, citation practices exist in the context of current regimes of evaluating science. While it may go unnoticed in daily writing practices, the act of including a single reference in a study is thus subject to value-based criteria internal to science (e.g., content, relevance, credit) and external to science (e.g., accountability, performance).

Accordingly, referencing is not a neutral act. Citations are a form of scientific currency, actively conferring or denying value. Citing certain sources—and especially citing them often—legitimises ideas, solidifies theories, and establishes claims as facts. References also create transparency by allowing others to retrace your steps. Referencing is thus a moral issue, an issue upon which multiple values in science converge. Citing competitors adds to their profiles, citing papers from a specific journal adds to its impact factor, citing supervisors or lab mates helps build your own profile, and citing the right papers helps establish your familiarity with the field. All of these translate into pressures on scientists to cite specific sources, from peers, editors, and others. Fong and Wilhite demonstrate the abundance of so-called coercive citation practices [1]. Also, citation-based metrics have proliferated as proxies for quality and impact over the years [2–4], only to be currently subjected to significant and highly relevant critique [5–8]. To cite well, or to reference responsibly, is thus a matter of concern to all scientists.

Here, I offer 10 simple rules for responsible referencing. Scientists as authors produce references, and as readers and reviewers, they assess and evaluate references. Through this symmetrical relationship to literature that all scientists share, they take responsibility for tying together all knowledge it contains. Producing and evaluating references are, however, distinct processes, warranting different responsibilities. Respecting this dual relationship researchers have with literature, the first six rules primarily refer to producing a citation and the responsibilities this entails. The second set of four rules refers to evaluating citations and the meaning they have or acquire once they have become part of a text.

## Rule 1: Include relevant citations

All scholarly writing requires a demonstration of the relevance of the questions asked, a display of the methods used, a rationale for the use of materials, and a discussion of issues relevant to the content of the publication. All of these are done, at least in large part, by including citations to relevant previous work. Omitting such references can wrongfully suggest that your own publication is the origin of an idea, a question, a method, or a critique, thereby illegitimately appropriating them. Citations identify where ideas have come from, and consulting the cited works allows readers of your text to study them more closely, as well as to evaluate whether your use of them is appropriate.

A single exception exists when facts, findings, or methods have become part of scientific or scholarly canon. There is no need to include a citation on the claim that DNA is built out of four bases, nor do you have to cite Kjell Kleppe or Kary Mullis every time you use PCR (neither do I right now). However, the decision as to when something truly becomes part of canon can be quite difficult and will include periods of adjustment (with irregular citation) and negotiation (on whether to cite or not).

## Rule 2: Read the publications you cite

Citation is not an administrative task. First, a single paper can be cited for multiple reasons, ranging from reported data to methods, and can be cited both positively and negatively in the literature. The only way to identify whether its content is relevant as support for your claim is to read it in full.

Second, the collection of citations included to support your work and argument is one of the elements from which your work draws credibility. The same goes for the citations you include to criticise, dispute, or disprove. As a consequence, a chain is only as strong as its weakest link. The quality of the publication you trust and upon which you confer authority codetermines the quality and credibility of your work. Citation rates, especially on the journal level, do not correspond well to research quality [9], and they conflate positive and negative citations, not distinguishing authority conferred or authority that is challenged. To cite meaningfully and credibly requires that you consult the content of a publication rather than whether others have cited it, as a criterion for citation.

## Rule 3: Cite in accordance with content

If, at some phase in the research, you have decided that a specific study merits citation, the issue of specifically how and where to cite it deserves explicit consideration. Mere inclusion does not suffice. Sources deserve credit for the exact contribution they offer, not their contribution in general. This may mean that you need to cite a single source multiple times throughout your own argument, including explanations or indications why.

A specific way to break Rule 3 is in the form of the so-called ‘Trojan citation’ [10]. The Trojan citation arises when a publication reporting similar findings to your own is cited in the context of a discussion of a minor issue, ignoring (sometimes deliberately) its key argument or contribution. By focussing on a trivial detail, the Trojan citation obscures the true significance of the cited work. As a consequence, it hides that your work is not as novel as it seems. As a questionable citation practice, a Trojan citation can be used to satisfy reviewers’ or editors’ requests to include a reference to a relevant paper. Alternatively, a Trojan citation may emerge unknowingly when (1) you are unaware of the content of a cited publication (not adhering to Rule 2 creates a very significant risk of being unable to follow Rule 3) or (2) disputes exist in the scientific community or among the authors on the contribution and/or quality of a scientific publication (in which case, Rule 4 will help).

## Rule 4: Cite transparently, not neutrally

Citing, even in accordance with content, requires context. This is especially important when it happens as part of the article’s argument. Not all citations are a part of an article’s argument. Citations to data, resources, materials, and established methods require less, if any, context. As part of the argument, however, the mere inclusion of a citation, even when in the right spot, does not convey the value of the reference and, accordingly, the rationale for including it. In a recent editorial, the *Nature Genetics* editors argued against so-called neutral citation. This citation practice, they argue, appears neutral or procedural yet lacks required displays of context of the cited source or rationale for including [11]. Rather, citations should mention assessments of value, worth, relevance, or significance in the context of whether findings support or oppose reported data or conclusions.

This flows from the realisation that citations are political, even though that term is rarely used in this context. Researchers can use them to accurately represent, inflate, or deflate contributions, based on (1) whether they are included and (2) whether their contributions are qualified. Context or rationale can be qualified by using the right verbs. The contribution of a specific reference can be inflated or deflated through the absence of or use of the wrong qualifying term (‘the authors suggest’ versus ‘the authors establish’; ‘this excellent study shows’ versus ‘this pilot study shows’). If intentional, it is a form of deception, rewriting the content of scientific canon. If unintentional, it is the result of sloppy writing. Ask yourself why you are citing prior work and which value you are attributing to it, and whether the answers to these questions are accessible to your readers.

## Rule 5: Cite yourself when required

In the context of critical discussions of citations and evaluations of citation-based metrics, self-citation has almost become a taboo. It is important to realise, though, that self-citation serves an important function by showing incremental iterative advancement of your work [12]. As a consequence, your previous work or that of the group in which you are embedded should be cited in accordance with all of the rules above. The amount of acceptable self-citation is very likely to differ between fields; smaller fields (niche fields) are likely to (legitimately) exhibit more.

This does not mean that self-citation is always unproblematic. For instance, excessive self-citation can suggest salami slicing, a publication strategy in which elements of a single study are published separately [13]. This questionable research practice, in tandem with self-citation, aims to inflate publication and citation metrics.

## Rule 6: Prioritise the citations you include

Many journals have restrictions on the number of references authors are allowed to include. The exact number varies per publisher, journal, and article type and can be as low as three (for a correspondence item in *Nature*). Even if no reference limit exists, other journals impose a word limit that includes references, effectively also capping the amount of references. Coping with these limits sometimes requires difficult decisions to omit citations you may feel are legitimate or even necessary. In order to deal with this issue and avoid random removal of references, all desired citations require prioritisation. A few rules of thumb, shown in Box 1, will help decisions on reference priority.

**Box 1: Reference prioritisation**

‘Ten simple sub-rules for prioritising references’ can help to facilitate prioritisation. In most cases, a subset of the 10 sub-rules will suffice. First, prioritise anew for each publication. Prioritisations cannot (easily) be copied from one study to another. Second, prioritise per section (e.g., introduction, methods, discussion), not across the entire paper. Different sections require different types of support. Third, for the introduction, prioritise reviews, allowing broad context for relevance and aim. Fourth, for the discussion, prioritise empirical papers, allowing detailed accounts of relative contribution. Fifth, prioritise reviewed over un- or prereviewed papers (e.g., editorials, preprints, etc.). Sixth, deprioritise self-citations. Seventh, limit the number of citations to support a specific claim, if necessary, to a single citation. Eighth, move methodological citations to supplementary (online) information. Ninth, in cases of equal relevance, prioritise citation of female first or last authors to help repair gender imbalances in science. Tenth, request the inclusion of additional references with the editors, arguing that you have used all of the previous nine sub-rules.

## Rule 7: Evaluate citations as the choices that they are

Research publications are not mere vessels of data or findings. They convey a narrative explaining why questions are worth asking, what their answers may mean, how these answers were reached, why they are to be trusted, and more. They also have a purpose in the sense that they will act as support for other studies to come. Each of the elements of their story is supported by links to other studies, and each of those links is the result of an active choice by the author(s) in the context of the goal they wish to achieve by their inclusion.

At the other end of the narrative, readers assess and evaluate the story constantly, asking whether it could have been told differently. The realisation that narratives can be told differently, supported by other citations to other prior work, does not disqualify them. Both the story and the choice of citations are political choices meant to provide the argument with as much power, credibility, and legitimacy the author(s) can muster. They are tailored to the audience the authors seek to convince: their peers. The choice to include or exclude a reference can only be evaluated in the context of that narrative and the role they play in it. Peritz has provided a classification of citation roles to assist this evaluation [14].

## Rule 8: Evaluate citations in their rhetorical context

Rhetorical strategies serve to convince and persuade. Narratives are but one of the tools that can be used to persuade audiences. Metaphors, numbers, and associations all feature in our research papers as tools to convince our readers. The genre of the scientific article has had centuries to evolve to incorporate many of them, with the goal of convincing readers that the author is right. Bazerman has literally written the book on this [15] and urges us to consider academic texts and their features as part of social and intellectual endeavours. Citations are a part of the social fabric of science in the sense that through citing specific sources, authors show their allegiance to schools of thought, communities, or, in the context of scientific controversies, which paradigm they consider themselves part of. Other rhetorical uses of citations include explicit citations to notable figures and their work, which can serve as appeals to authority, while long lists of citations can serve as proxies for well-studied subjects.

Consider the following: Authors can describe a field as well-studied and include three references—X, Y, and Z—as support for their claim. Alternatively, they can argue that a field is understudied but that three exceptions exist, i.e., X, Y, and Z. Understanding the value attributed to X, Y, and Z in that particular text requires assessment of the rhetorical strategies of the author(s).

## Rule 9: Evaluate citations as framed communication

Authors use words to accomplish things and, in service of those goals, position their work and that of others. They frame prior work in a very specific way, supporting the arguments made. We all do. The positioning of X, Y, and Z either as the norm or as exceptions, as shown in Rule 8, is an example of framing. It is important to recognise such framing and that X, Y, and Z acquire meaning in the text as the result of the frame. There is no frameless communication, as Goffman [16] demonstrated. All messages and texts contain and require a frame—a structure of definitions and assumptions that help organise coherence, connections, and, ultimately, meaning—or in other words, a perspective on reality.

As a result, a citation is not a neutral line drawn between publications A and B. Rather, the representation of cited article A only acquires meaning in the context of citing in article B. Article A can be framed differently when cited in work B or C. It can be framed as innovative in B or dogmatic in C. Framing usually is not lying or deceiving; it is a normative positioning of evidence in context. Hence, a citation is a careful translation of a source’s relevant elements, which acquire meaning in that context only.

An important consequence of this is that merely counting citations of article A in the literature does not inform us of the value (or many types of value or lack thereof) of article A to the scientific community. This point also appears as the first principle in the Leiden Manifesto, which argues that quantitative metrics can only support qualitative metrics (i.e., reading with an attentive eye for politics, rhetoric, context, and frame—or as adhering to Rules 7–9). The Leiden Manifesto was published by bibliometricians and scholars of research evaluation following the 2014 conference on Science and Technology Indicators in Leiden, the Netherlands. It warns against the abuse of, among other things, citation-based research metrics [9].

## Rule 10: Accept that citation cultures differ across boundaries

Despite critiques of the system, science is organised in such a way that citations continue to act as a currency that is represented as being universal [4]. However, citation practices are, for the most part, local practices, whether local to laboratories or department or local to disciplines. The average number of citations per paper differs between disciplines, and the way that citations are represented in the text and the value of being cited also differ radically [17]. What counts as proper citation practice in molecular biology—for instance, the inclusion of multiple references following a statement—is considered unacceptable in research ethics or science policy, in which single references require paragraphs of contextualisation and translation (see Rule 9). When reading a paper from an adjacent discipline, respect its different norms and conventions for responsible referencing and proper citation. If you are cited by a scientist from another discipline, assess that act as existing in a (however slightly) different citation culture.

## References

[1] Fong E, Wilhite A. Authorship and citation manipulation in academic research. PloS ONE. 2017;12(12):e0187394 doi: 10.1371/journal.pone.0187394 29211744 10.1371/journal.pone.0187394 PMC5718422

[2] Feller I. Performance measurement and the governance of American academic science. Minerva. 2009;47(3):323.

[3] Garfield E, Merton R. Citation indexing: Its theory and application in science, technology, and humanities: New York: Wiley; 1979.

[4] Wouters P. The citation culture. Amsterdam: University of Amsterdam; 1999.

[5] Dahler-Larsen P. Constitutive effects of performance indicator systems Dilemmas of engagement: Evaluation and the new public management. Emerald Group Publishing Limited; 2007 p. 17–35.

[6] De Rijcke S, Wouters P, Rushforth A, Franssen T, Hammarfelt B. Evaluation practices and effects of indicator use—a literature review. Research Evaluation. 2016;25(2):161–9.

[7] Bornmann L, Daniel H-D. What do citation counts measure? A review of studies on citing behavior. Journal of documentation. 2008;64(1):45–80.

[8] Müller R, de Rijcke S. Exploring the epistemic impacts of academic performance indicators in the life sciences. Research Evaluation. 2017;26(3):157–68.

[9] Hicks D, Wouters P, Waltman L, De Rijcke S, Rafols I. Bibliometrics: the Leiden Manifesto for research metrics. Nature. 2015;520:429–31. doi: 10.1038/520429a 25903611 10.1038/520429a

[10] Shaw D. The Trojan Citation and the “Accidental” Plagiarist. Journal of Bioethical Inquiry. 2016;13(1):7–9. doi: 10.1007/s11673-015-9696-7 26780105 10.1007/s11673-015-9696-7

[11] Nature Genetics. Neutral citation is poor scholarship. Nature Genetics. 2017;49:1559 doi: 10.1038/ng.3989 29074946 10.1038/ng.3989

[12] Hyland K. Self-citation and self-reference: Credibility and promotion in academic publication. Journal of the Association for Information Science and Technology. 2003;54(3):251–9.

[13] Jackson D, Walter G, Daly J, Cleary M. Multiple outputs from single studies: Acceptable division of findings vs. ‘salami’ slicing. Journal of clinical nursing. 2014;23(1–2):1–2. doi: 10.1111/jocn.12439 24313937 10.1111/jocn.12439

[14] Peritz B. A classification of citation roles for the social sciences and related fields. Scientometrics. 1983;5(5):303–12.

[15] Bazerman C. Shaping written knowledge: The genre and activity of the experimental article in science. Madison, WI: University of Wisconsin Press; 1988.

[16] Goffman E. Frame analysis: An essay on the organization of experience.Cambrdige, MA: Harvard University Press; 1974.

[17] Aksnes D, Rip A. Researchers’ perceptions of citations. Research Policy. 2009;38(6):895–905.

